# HSPIR: a manually annotated heat shock protein information resource

**DOI:** 10.1093/bioinformatics/bts520

**Published:** 2012-08-24

**Authors:** Ratheesh Kumar R., Nagarajan N. S., Arunraj S. P., Devanjan Sinha, Vinoth Babu Veedin Rajan, Vinoth Kumar Esthaki, Patrick D’Silva

**Affiliations:** Department of Biochemistry, Indian Institute of Science, Bangalore 560012, Karnataka, India

## Abstract

**Summary:** Heat shock protein information resource (HSPIR) is a concerted database of six major heat shock proteins (HSPs), namely, Hsp70, Hsp40, Hsp60, Hsp90, Hsp100 and small HSP. The HSPs are essential for the survival of all living organisms, as they protect the conformations of proteins on exposure to various stress conditions. They are a highly conserved group of proteins involved in diverse physiological functions, including *de novo* folding, disaggregation and protein trafficking. Moreover, their critical role in the control of disease progression made them a prime target of research. Presently, limited information is available on HSPs in reference to their identification and structural classification across genera. To that extent, HSPIR provides manually curated information on sequence, structure, classification, ontology, domain organization, localization and possible biological functions extracted from UniProt, GenBank, Protein Data Bank and the literature. The database offers interactive search with incorporated tools, which enhances the analysis. HSPIR is a reliable resource for researchers exploring structure, function and evolution of HSPs.

**Availability:** http://pdslab.biochem.iisc.ernet.in/hspir/

**Contact:** patrick@biochem.iisc.ernet.in

**Supplementary information:** Supplementary data are available at *Bioinformatics* online.

## 1 INTRODUCTION

Heat shock proteins (HSPs) are a specialized group of proteins robustly synthesized in all living organisms in response to various conditions of stress, including elevated temperatures. HSPs are critical for cell survival both constitutively and in times of stress to ensure proper folding of non-native states of proteins (Bukau *et al.*, 2006; Parsell and Lindquist, 1993). Based on their nature of functions and molecular mass, HSPs are classified broadly into six major families, namely, Hsp70, Hsp40 (J-proteins), Hsp60 (chaperonins), Hsp90, Hsp100 (Clp proteins) and small HSPs. (Lund 2001; Kampinga and Craig, 2010)

They function cooperatively by forming an intricate molecular network, thereby maintaining the overall cellular protein homeostasis (Lund, 2001). Their diversified nature and vast repertoire of functions have generated a significant interest to deduce an intricate cellular chaperone network and functional crosstalk among major families of HSPs.

Presently, ‘cpnDB’ (Hill *et al.*, 2004) database exists but contains data only for Hsp60 family. Heat shock protein information resource (HSPIR) provides a comprehensive collection of information on six major HSP families across various genomes, with detailed subclassification based on their domain, structural organization and localization. HSPIR also includes sequences that are not yet annotated in UniProt. Additionally, HSPIR offers various tools like BLAST (Altschul *et al.*, 1997) for homology search, CLUSTALW (Larkin *et al.*, 2007) for multiple sequence alignment, Archaeopteryx (Han and Zmasek, 2009) for phylogenetic tree visualization and manipulation and Jmol (http://www.jmol.org/) structural viewer. The database currently holds ∼10 000 hand-curated entries from six kingdoms, covering all the major model organisms and 295 3D structures.

## 2 DATA RETRIEVAL AND CURATION

We did an extensive literature survey to retrieve names, nomenclature, functions and structural information of HSPs using the PubMed query system. With this knowledge, we created a comprehensive list of standard names and alternative names for each HSP family. Structures and their corresponding sequences of HSPs were retrieved from Protein Data Bank (PDB). The aforementioned generated data were used for keyword and sequence search against SwissProt (Boeckmann *et al.*, 2003). These data sets were then filtered to include sequences that belong to protein existence level 1 or 2 (evidence at protein level or evidence at the transcript level, respectively). Sequences with domains that are partial in length or missing any functional motifs were discarded. Using these initial data sets as seed sequences (refer Supplementary Table S1), position-specific scoring matrix (PSSM) was created for each family of HSP. Organism-specific PSI-BLAST was performed using the PSSM with an e-value cut-off of 0.0001 against the NCBI non-redundant protein sequence database (Altschul *et al.*, 1997; Benson *et al.*, 2011) to populate HSPIR. Extreme care was taken to remove the duplicated and highly truncated sequences from the data sets. These collated data sets were then manually curated by taking single sequence at a time and using different database search methods to annotate structural and functional information (Supplementary Figure S1).

We used a wider collection of the protein family databases such as NCBI CDD (Marchler-Bauer *et al.*, 2011), Pfam (Finn *et al.,* 2010), InterPro (Hunter *et al.,* 2009) and SMART (Letunic *et al.*, 2009) to identify domain architecture and associated functional motifs of HSPs. The secondary structural assignments were done using PSIPRED Version 3.2 (McGuffin *et al.*, 2000), and subcellular localization and signal peptide regions were predicted using TargetP (Emanuelsson *et al.,* 2007; Nielsen *et al.,* 1997), WolfPsort (Horton *et al.,* 2007) and Psort (Nakai and Horton, 1999).

Taxon information of each organism was obtained from NCBI Taxonomy database (Benson *et al.,* 2011). Gene ontologies were inferred from UniProtKB (The UniProt Consortium, 2012). Experimentally determined 3D structures were retrieved from the PDB (Berman *et al.,* 2000). Available literature references were generated from PubMed; the cross-references and identifiers of external databases were also imported.

## 3 DATABASE IMPLEMENTATION

HSPIR is built using open source MySQL database and interfaced with server-side PHP scripts. The Web interface uses dynamically generated HTML pages supported by JavaScript and CSS to provide an interactive environment for public access. Perl-CGI scripts have been used to compile the BLAST, CLUSTALW and hidden Markov model search features (Eddy, 1998).

## 4 DATABASE INTERFACE AND VISUALIZATION

HSPIR is a user-friendly Web resource in which the homepage provides a model of functional networks of six major HSP families (Supplementary Figure S2). Each of them is mapped with a dedicated Web page explaining their structure, domain organization, classification and physiological significance with diagrammatic illustrations.

### 4.1 Search features

HSPIR incorporates four different search features. Basic keyword search allows finding of HSPs based on their names (includes gene names, standardized and synonymous names), families, identifiers (HSPIR and external) and classifications. The advanced search is our key feature, which narrows the search criteria for specific and better results. Users are able to refine their search using the combined search method with logical and relational operators dynamically organized on the page. The data retrieval can be further streamlined using other specialized query tools such as genome-wide search and domain-based search. These tools can query database independently to retrieve records based on a specific genome, specified combination of domains.

### 4.2 Results

Results of all the search tools are presented in the form of a paginated table. Protein records can be viewed by clicking the accession ID, or have been added to the HSPIR cart for downloading and further analysis. Individual protein records comprise names and lineage, classification, sequence information, domains and motifs, structures, ontologies, references, cross-references, external links to different databases and, finally, the protein record information.

### 4.3 Sequence comparison

BLAST stand-alone package implemented in the database allows the users to search for a query protein against HSPIR database and identify similar HSP sequences. Comparing of multiple sequences can be done using CLUSTALW, and the tree is visualized using Archaeopteryx.

### 4.4 HSP identification

The HSP identification tool allows the user to identify and classify unknown sequences into a particular HSP family. The user-provided sequence is scanned against predefined HSP libraries of profiles created from a set of validated seed sequences (Supplementary Table S1).

## 5 SUMMARY AND FUTURE PRESPECTIVES

The scope of HSPIR is to provide a dedicated resource for HSPs with functional annotations. The interactive search features with collated information provided in the database will allow researchers to perform comparative analysis and explore additional physiological functions of HSPs in different species, which was not well appreciated previously. Moreover, the data in the HSPIR will be checked for updates weekly, using PHP scripts and parsers scheduled by crontab. These updated records will be reviewed and uploaded by the curation team. The future perspective is to incorporate HSP information for additional genomes, with a special emphasis on pathogenic species. We will include other specialized chaperones like disulfide isomerases, accessory proteins such as nucleotide exchange factors, prefoldins and HSP90 co-chaperones.

## Supplementary Material

Supplementary Data
