# Supplementary material for: HSPIR: a manually annotated heat shock protein information resource
Source: Bioinformatics. 2012 Aug 24;28(21):2853–5. doi: 10.1093/bioinformatics/bts520 (PMC3476333; doi:10.1093/bioinformatics/bts520)
Supplement: Supplementary Data [file supp_28_21_2853__index.html]

HSPIR: a manually annotated heat shock protein information resource — Supplementary Data 

# HSPIR: a manually annotated heat shock protein information resource

## Supplementary Data

files

**Files in this Data Supplement:**

- Supplementary Data - pdf file
- Supplementary Data - xls file
