# Supplementary material for: HSPIR: a manually annotated heat shock protein information resource
Source: Bioinformatics. 2012 Aug 24;28(21):2853–5. doi: 10.1093/bioinformatics/bts520 (PMC3476333; doi:10.1093/bioinformatics/bts520)
Supplement: Supplementary Data [file supp_bts520_Supplementary_Data.pdf]

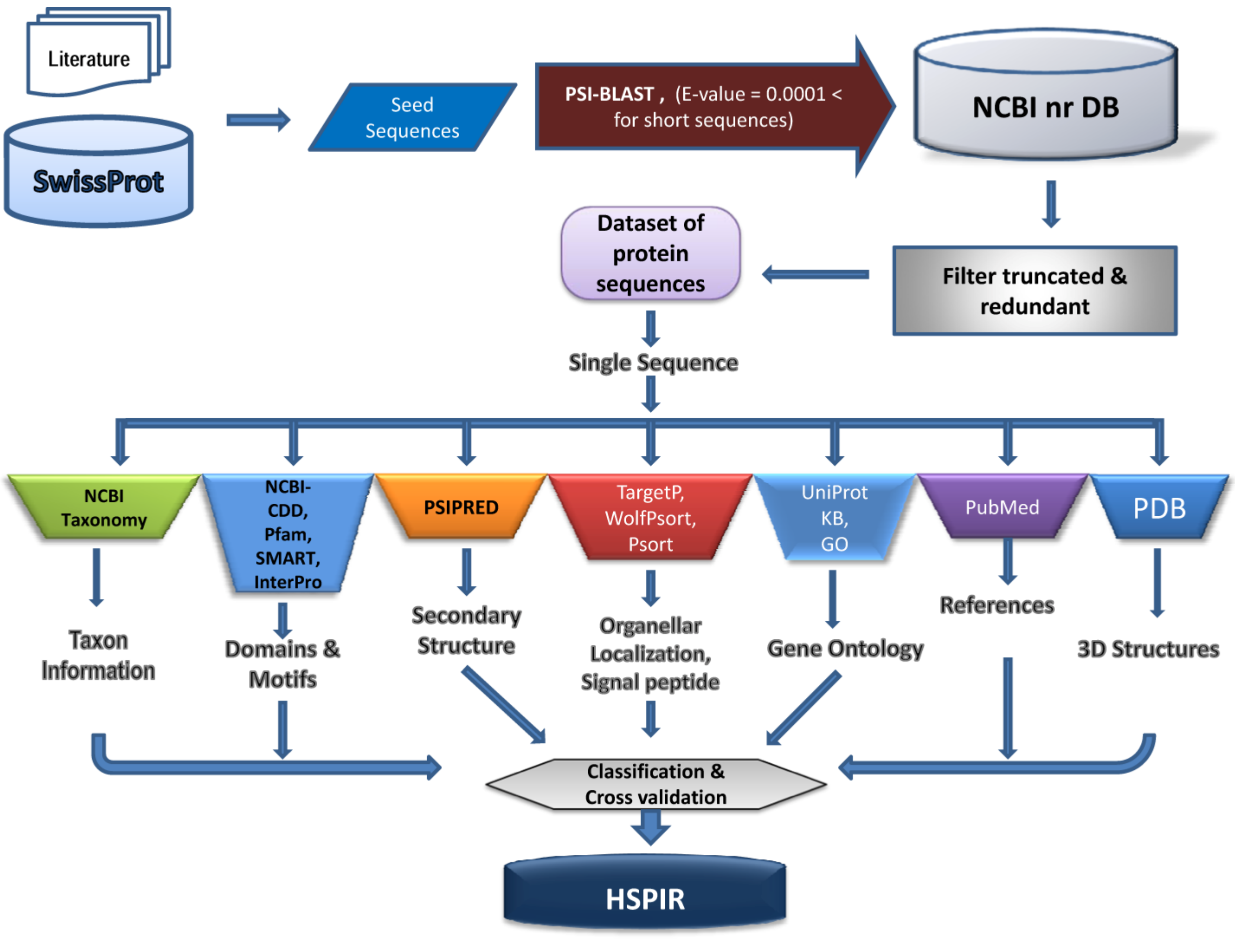

Supplementary Figure S1: Schematic representation of the data retrieval and curation process implemented in HSPIR database creation

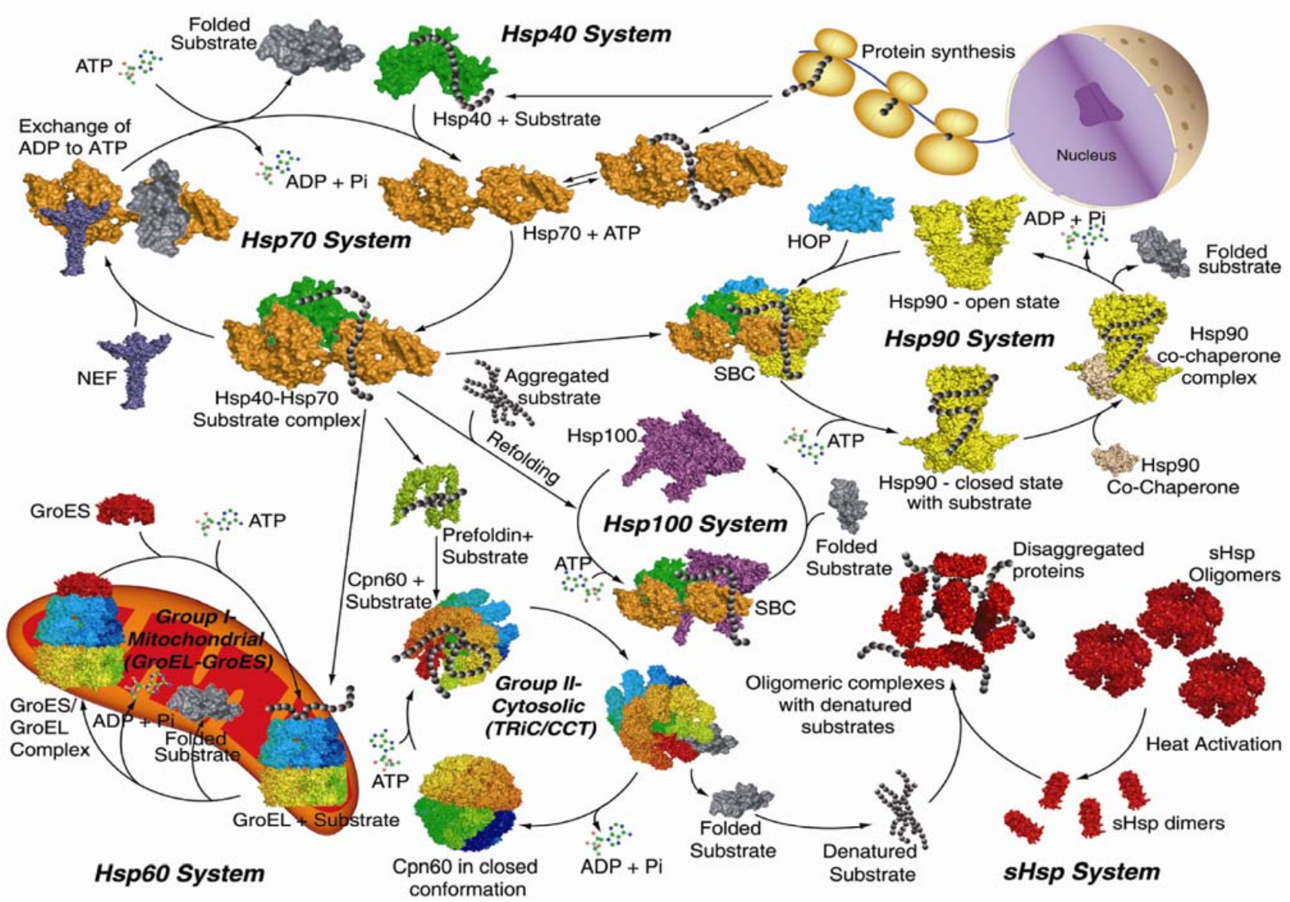

Supplementary Figure S2: A functional HSP family network required for the maintenance of cellular protein quality control. The 3D models for the protein structure were created with PyMOL 1.3 using the following PDB codes: 1XAO (Hsp40/J-protein), 2KHO (Hsp70), 1DKG (NEF), 1WE3 (GroEL/GroES), 3IYF (Cpn60-open state), 3LOS (Cpn60-closed state), 2ZDI (Prefoldin), 2BYU (sHsp), 1QVR (Hsp100), 2OIU (Hsp90-open state), 2CG9 (Hsp90-closed state), 3ESK (HOP). Abbreviations: NEF- Nucleotide Exchange Factor, HOP- Hsc70/Hsp90 Organizing Protein, SBC- Substrate Binding Complex.
